# A Survey of Naturally-Occurring Steroid Hormones in Raw Milk and the Associated Health Risks in Tangshan City, Hebei Province, China

**DOI:** 10.3390/ijerph15010038

**Published:** 2017-12-26

**Authors:** Xueyin Qu, Chuanyou Su, Nan Zheng, Songli Li, Lu Meng, Jiaqi Wang

**Affiliations:** 1 Ministry of Agriculture-Laboratory of Quality and Safety Risk Assessment for Dairy Products (Beijing), Institute of Animal Science, Chinese Academy of Agricultural Sciences, Beijing 100193, China; qu_xueyin@163.com (X.Q.); suchuanyou2010@126.com (C.S.); Lisongli@caas.cn (S.L.); marilyn0307@163.com (L.M.); wang-jia-qi@263.net (J.W.); 2 State Key Laboratory of Animal Nutrition, Institute of Animal Science, Chinese Academy of Agricultural Sciences, Beijing 100193, China; 3 College of Animal Science and Technology, Shihezi University, No. 4, the North Road, Shihezi 832002, China

**Keywords:** raw milk, steroid hormone, progesterone, HPLC-MS/MS

## Abstract

In recent years, high levels of hormone residue in food, capable of damaging the health of consumers, have been recorded frequently. In this study, 195 raw milk samples were obtained from Tangshan City, China, and the concentrations of 22 steroid hormones were measured by high-performance liquid chromatography-tandem mass spectrometry (HPLC-MS/MS). Cortisol was detected in 12.5% of raw milk samples (mean 0.61 µg/kg; range: <limit of quantification (LOQ)–0.94 µg/kg). Progesterone was detected in 85.9% of raw milk samples (mean 5.12 µg/kg; range: <LOQ–9.04 µg/kg). The concentration of cortisol present in milk was lower than the maximum residue limit defined in Japan (10 µg/kg). Children aged between one and five years were expected to be the at-risk population when exposed to detectable steroid hormone concentrations. Based on the mean and maximum concentrations of progesterone detected in milk, the contribution was 0.85% and 0.9%, and 1.48% and 1.6% of the acceptable daily intake for boys and girls, respectively. These results imply that the concentrations of steroid hormones present in raw milk should not present a health risk for young children.

## 1. Introduction

Steroid hormones have been extensively used in animal husbandry because they can promote the growth of animals and improve feed conversion efficiency [1]. Steroid hormones include androgens, estrogens, glucocorticoids, and progestogens and related synthetic compounds [2]. In veterinary medicine, steroid hormones are used in cows for therapeutic and zootechnical purposes [3]. Hormones in milk can be classified in two groups: natural hormones and synthetic hormones (for example, some estrogenic hormones) [1,4]. Hormones in milk originate from the blood flow and are secreted into the milk through an active transport within the mammary gland. Some hormones can also be synthesized by the mammary gland and excreted into the milk [1,5,6].

There is concern about the presence of hormone residues in food because of their endocrine-disrupting effects in humans [6,7]. Some reports have indicated that the steroid hormones in food derived from animals have the potential to be toxic and carcinogenic, and their residues may be relevant to a variety of diseases including cancer of the breasts, ovaries and prostate [8,9]. Several animal studies have shown that exposure to steroid hormones can produce adverse effects on male and female reproductive development. Female rats exposed prenatally to testosterone have altered reproduction later in life and no nipple development (i.e., masculinized mammary morphology), suggesting that gestational exposure to testosterone severely inhibits the normal development of females, rendering them unable to nurse their offspring [10]. In the male rat, exposure to estradiol was reported to induce morphological lesions and spermatogenic arrest [11]. Besides, the continued and massive intake of estrone may enhance tissue deposition and lead to obesity [12]. The accidental ingestion of food containing high levels of testosterone, can lead athletes to be disqualified, for testosterone was listed as one of the prohibited substances by the World Anti-Doping Agency [13]. In recent years, a number of studies have identified prolactin and steroids in dairy products, including estrogens, progesterone, glucocorticoids and androgens. And recently it is found that these compounds may have significant biological effects even at very low doses. Special concern should be paid to the effects [14]. In the report by Yang et al. (2009), the concentrations of 17α-hydroxyprogesterone, testosterone, progesterone, 4-androstene-3, 17-dione, and estradiol were found to be 0.11–6.10 μg/kg in milk [2]. Chen et al. (2014) determined the concentrations of the natural steroid hormones estrone, 17α-estradiol, 17β-estradiol, and estriol in commercial milk samples, finding them to be 0–146.12 ng/L, 0–70.12 ng/L, 0–31.85 ng/L and 0–2.18 ng/L, respectively [15]. The identification of hormones in milk that have the potential to disrupt the physiological function of endocrine systems has raised more concern.

Milk and milk products are an important component of the human diet, especially in Western countries, but also more recently in Asia [14]. The annual consumption per capita, converted into a quantity of raw milk, was 36 kg during 2015 in China [16]. Although the use of steroids in food-producing animals for therapeutic purposes is regulated or banned in China, and a detectable residue is not permitted in animal feed [17], naturally-occurring hormones can be present at a wide range of concentrations in dairy products, depending on factors including physiologic conditions, diet composition, breed, and veterinary drug administration [14,18].

The purpose of this study was to quantify the naturally-occurring steroid hormones in raw milk in Tangshan, which is an important milk-producing city in China and has a population of nearly 7.84 million. Glucocorticoids and sex hormones (androgens, estrogens, and progestogens) in raw milk were determined using high-performance liquid chromatography-tandem mass spectrometry (HPLC-MS/MS), and the exposure of children, as the most at-risk population consuming milk, was estimated for each.

## 2. Materials and Methods

### 2.1. Sampling

**Figure 1:**
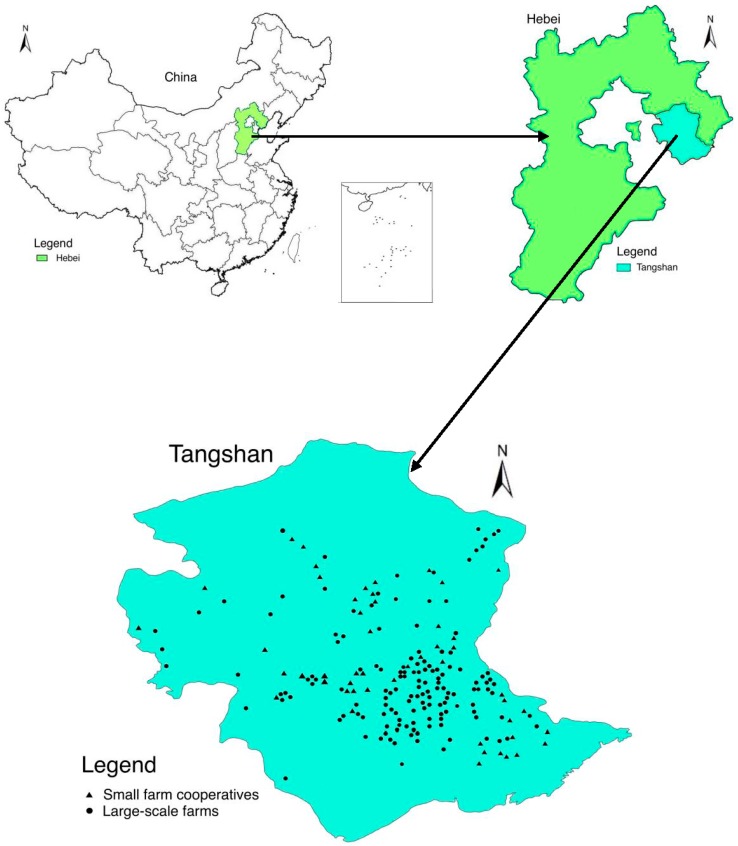
Sampling sites of raw milk from Tangshan, Hebei, China.

Between May and August 2014, raw milk samples were collected from 195 milk stations in Tangshan City, Hebei Province, China. Sampling sites of raw milk are shown in Figure 1. According to their ownership, the 195 milk stations can be classified into two types: those owned by large-scale farms (141) and small farm cooperatives (54). The raw milk was collected from milk tanks at the milk stations. After stirring the milk in each holding tank, 100 mL of raw milk sample was taken from the upper third, 100 mL from the middle third, and 100 mL from the lower third. A total of 300 mL milk from each tank was mixed and 100 mL of it was taken out and stored at −20 °C until analysis. 

### 2.2. Chemicals and Materials

Carbinol, dichloromethane, formic acid, and acetonitrile were all chromatographically pure; and were purchased from Merck (Darmstadt, Germany). Acetic acid (analytically pure), sodium acetate (analytically pure), and β-glucuronidase/arylsulfatase were purchased from Merck (St. Louis, MO, USA). ENVI-carb solid phase extraction (SPE) cartridges (500 mg, 6 mL; graphitized carbon black (GCB) cartridge) were supplied by Supelco (Bellefonte, PA, USA). Amino SPE columns (500 mg, 6 mL) were from Waters (Milford, MA, USA). Standards were obtained from Dr. Ehrenstorfer Gmbh (Augsburg, Germany) and CDN Isotopes (Pointe-Claire, QC, Canada). Internal standards, including 17β-boldenone-*d*_3_, testosterone-3,4-^13^C_2_, methyltestosterone-*d*_3_, 16β-OH-stanozolol-*d*_3_, megestrol acetate-*d*_3_, progesterone-*d*_9_, cortisol-*d*_3_, estradiol-3, 4-^13^C_2_, estrone-2,4-*d*_2_, diethylstilbestrol-*d*_6_, and dienestrol-*d*_2_ were obtained from CDN Isotopes (Pointe-Claire, QC, Canada). Standard stock solutions of individual and internal standards were prepared at 1.0 mg/mL in methanol and stored at −20 °C. The internal standard solutions were combined and diluted with methanol to a concentration of 100 µg/L for use as a working solution. Spiking and calibration mixtures at various concentrations were prepared by combining stock and internal standard working solutions with 50% methanol. The concentrations of internal standards in all calibration mixtures and final sample solutions were 10 µg/L. Deionized water was obtained from a Milli-Q Academic (Millipore, Bedford, MA, USA) purification system.

### 2.3. Preparation of Samples

The detection method of steroid hormones in this text was performed according to GB/T 21981-2008 [19]. Five gram milk samples were transferred to 50 mL centrifuge tubes and spiked with 100 µL internal standard working solution (100 µg/L). Ten milliliters of sodium acetate and acetic acid buffer solution (pH 5.2) was added and the mixture was homogenized with a vortex mixer for 5 min. Then, 100 µL β-glucuronidase/arylsulfatase was added to each sample and the solution was incubated for 12 h at 37 °C. After the solution had been cooled to room temperature, 25 mL methanol was added and ultrasonic extraction performed for 30 min. The samples were then centrifuged for 10 min at 10,000 r/min at 4 °C. The upper layer was transferred to 50 mL centrifuge tubes and diluted with water up to 50 mL final volume. ENVI-Carb SPE cartridges and amino columns were then used to clean this extract. The solution was passed through preconditioned GCB cartridges at 2–3 mL/min, followed by 3 mL dichloromethane-methanol (70/30, *v*/*v*), and 3 mL water. After the samples were loaded, the GCB cartridges were dried using a vacuum pump. Amino columns were preconditioned with 4 mL DCM–methanol (7/3, *v*/*v*) and placed beneath the GCB cartridges. The target compounds were eluted with 6 mL dichloromethane–methanol (7/3, *v*/*v*) and collected. The GCB cartridges were then removed and the amino columns washed with 2 mL dichloromethane–methanol again. Finally, the extracts were dried under nitrogen and dissolved in 1 mL methanol–water (1/1, *v*/*v*).

### 2.4. HPLC-MS/MS

Chromatographic analysis was performed using an Acquity HPLC system equipped with an Acquity HPLC BEH C18 column (1.7 μm particle size, 100 mm × 2.1 mm, Waters, Milford, MA, USA) maintained at 40 °C. For androgens, glucocorticoids and progestogens, the mobile phase consisted of water containing 0.1% formic acid (solvent A) and methanol (solvent B) with the gradient program: 50–64% solvent B from 0 to 8 min; 64–84% solvent B from 8 to 11 min; 84–100% solvent B from 11 to 12.5 min; 100% solvent B at 14.5 min; 100–50% solvent B from 14.5 to 15 min; and 50% solvent B at 17 min. For estrogens, the mobile phase consisted of water (solvent A) and acetonitrile (solvent B) with the gradient program: 35–50% solvent B from 0 to 4 min; 50–100% solvent B from 4 to 4.5 min; 100% solvent B at 5.5 min; 100–35% solvent B from 5.5 to 5.6 min; and 35% solvent B at 9 min. The flow rate was 0.3 mL/min, and the injection volume was 10 μL.

Mass spectrometric analysis was performed using a Waters Acquity TQSTM Micromass Quattro Ultima triple-quadrupole MS quadrupole equipped with an electrospray ion (ESI) source (Micromass, Manchester, UK). The instrument was operated using a capillary voltage of 3.5 kV in positive mode for the detection of androgens and progestogens, and a capillary voltage of 3.0 kV in negative mode for the detection of glucocorticoids and estrogens. The source and vaporizer temperatures were set to 100 °C and 450 °C, respectively. The pressure of the collision chamber was 0.31 Pa. Optimized instrumental conditions are shown in Table 1.

### 2.5. Method Validation

Quantification of 22 steroids was performed using a linear regression analysis at concentrations of 0.5–50 μg/L, plotting the ratio of the peak areas for each compound and the internal standard (10 μg/L). The European Commission Decision 2002/657/EC suggests setting up a series of experiments to estimate recovery [20]. The recovery of the analytes was evaluated by spiking milk samples with three concentrations of the 22 steroid hormones: low (1 μg/kg), medium (2 μg/kg), and high (5 μg/kg). The limit of quantification (LOQ) of the analytes was estimated by determining the signal-to-noise ratio (S/N) at 10.

### 2.6. Exposure Assessment

Exposure assessment was performed for all the consumers and separately for children aged between one and five years using the mean concentrations of steroid hormones obtained from the 195 raw milk samples. The ingestion levels for children were obtained from the Child-Specific Exposure Factors Handbook published by the Environmental Protection Agency [21]. For children, 300 mL is the daily level of intake advised by the Dietary Guidelines for Chinese Residents [22]. The body weight used for the children were those published by the World Health Organization (WHO) [23]. The WHO derived an acceptable daily intake (ADI) of 30 μg/kg body weight for progesterone on the basis of a lowest observed effect level (LOEL) of 200 mg/day (3.3 mg/kg body weight) to see changes in the uterus [24]. All experimental procedures for this study were approved by the ethics committee of Chinese Academy of Agricultural Sciences.

## 3. Results and Discussion

### 3.1. Recovery and LOQ of Steroid Hormones

The results for LOQ and recovery are shown in Table 2. The LOQs for trenbolone, boldenone, nandrolone, methandriol, megestrol acetate, chlormadinone acetate, estradiol and estriol were 1 µg/kg; for diethylstilbestrol, oestrone and prednisone 2 µg/kg, and 0.4 µg/kg for the other steroids. The recovery values were between 70.3% and 92.5%, which is in accordance with the recommended recovery (70–110%) using the quantitative method published by the European Commission [21]. The calibration curve for the steroids was linear and showed a high correlation with the amount of analyte injected, with the r^2^ being >0.999.

### 3.2. Concentrations of Steroids in Raw Milk

The presence of 22 steroids in raw milk is recorded in Table 3. Only cortisol and progesterone were detected using the assay described. In the raw milk, 24 (12.3%) and 165 (84.6%) samples had detectable levels (i.e., above the LOQ) of cortisol and progesterone, respectively. The mean concentrations of cortisol and progesterone were 0.61 µg/kg (range: <LOQ–0.94 µg/kg) and 5.12 µg/kg (range: <LOQ–9.04 µg/kg), respectively.

Cortisol, the principal glucocorticoid, is transported from the blood into the mammary gland of cows [14]. Yang et al. (2009) reported that the cortisol concentration in milk is 0.05–1.25 µg/kg and the mean concentration 0.43 µg/kg, which are similar to those measured in this study (mean 0.61 µg/kg) [2]. However, Alexandrova and Mach (1983) reported the normal concentration of cortisol in cow’s milk to be 8–18 µg/L [25]. This difference may reflect individual differences among cows [26]. Moreover, Romero et al. (2015) and Verkerk et al. (1998) suggested that milk cortisol content was influenced by the response to acute stressors in lactating cows and goats [27,28]. In addition, Sgorlon et al. (2015) found that breed is a factor affecting milk cortisol concentration [18]. The use of cortisol in food-producing animals for therapeutic purposes is banned in the European Union (EU) [29]. However, China, the EU and the Codex Alimentarius Commission (CAC) do not set limits for cortisol residues in milk and other dairy products [17,29,30]. Japan has established a maximum residue limit for cortisol of 10 µg/kg [31].

Ginther et al. (1974) showed that milk progesterone varies between 15.1–26.2 μg/L during days 30–210 of pregnancy [32] and Regal et al. (2012) reported that the mean levels of progesterone were 0.082 and 0.824 μg/L in raw milk from non-pregnant and pregnant cows, respectively [33]. In milk, progesterone can be endogenous or exogenous in origin. Progesterone naturally occurs in milk at a concentration that varies significantly with the reproductive cycle and depends on species, sex, age, and physiologic status, with the lowest concentration being present during estrus (<0.2–0.92 μg/L) and the highest during the luteal phase (0.2–30 μg/L) and pregnancy (20–35.7 μg/L) [3,34]. The maximum value measured in our study was 9.04 μg/L, which falls within the normal range of progesterone concentration in raw milk. Progesterone is also administered exogenously to cows and mares for therapeutic and zootechnical purposes, in which it regulates oocyte maturation, ovulation, myometrial quiescence, mammary gland growth, and endometrial enzymes [3]. The EU Commission regulation No. 37/2010 states that progesterone should only be used for intravaginal therapeutic or zootechnical use, and therefore no maximum residue limit (MRL) is required [29].

Milk consumption has previously and controversially been designated a risk factor for cancer [14] because of the presence of estrogens. However, in this study, estrogens were undetectable in raw milk samples from Tangshan City. In the study by Chen et al. (2014), the concentrations of estrone, estradiol, and estriol in milk were 0–146.12 ng/L, 0–70.12 ng/L, and 0–2.18 ng/L, respectively [15]. This difference in findings might relate to the presence of naturally-occurring estrogens in milk being determined by the age and physiological condition of the animals [35,36].

To ensure public health, many countries have established MRLs for hormones in milk. Regulations in China a set MRLs for two steroids, betamethasone and dexamethasone [37]. CAC set MRLs for only one steroid [30]. In the EU and Japan, MRLs are set for four and nine steroids, respectively [29,31]. In addition, some must be undetectable in milk in China and Japan, including progesterone, androgen, estrogen, and estrogen-like compounds [17,31]. The MRLs and the identity of the steroids that are apparently undetectable are shown in Table 4.

### 3.3. Risk Assessment for the Presence of Cortisol and Progesterone in Milk

Cortisol is released in response to stress and low blood glucose concentration, and its functions are to increase blood sugar through gluconeogenesis and to modulate the immune system. Only a small amount of glucocorticoid is transferred from plasma into milk [5]. The basic chemical information of cortisol is not supplied in the WHO database. In addition, Xu et al. (2009) found no significant differences in cortisol content between bovine and human colostrum [26]. Therefore, the cortisol present in bovine milk may be deduced to have no adverse effects in children.

The exposure of children aged between one and five years was estimated in this study, because this group represents the most susceptible population. The data obtained are shown in Table 5 and Table 6. Based on the mean concentration of progesterone detected, the exposure levels for boys and girls were 0.092–0.252 μg/kg body weight/day and 0.093–0.271 μg/kg body weight/day, respectively. When the maximum concentration was used in the calculation, the exposure levels for boys and girls were 0.163–0.455 μg/kg body weight/day and 0.164–0.480 μg/kg body weight/day. The exposure to progesterone and age show an inverse relationship. The exposure level for girls was larger than boys, because the body weight of the girls was less than that of the boys. Young children were the most exposed group, but milk only contributed a maximum of 1.48% and 1.60% of the ADI for boys and girls, associated with a low dietary exposure to bovine milk.

According to reports by the Joint FAO/WHO Expert Committee on Food Additives (JECFA), the amount of exogenous progesterone that humans are exposed to through ingestion of tissue from treated animals is biologically insignificant, and is incapable of having a biologic effect in humans [3]. Our results indicate that the exposure due to milk consumption should not be associated with adverse effects in the population of Tangshan, China.

Few studies have reported the natural occurrence of steroid hormones in raw milk, although several have described methods for the detection of a variety of steroids in milk [1,2,33]. This study has shown that the level at which progesterone was detected would not result in a hazard for children and the maximum level of cortisol in raw milk was lower than the MRL specified in Japan (10 μg/kg).

## 4. Conclusions

A total of 195 samples of raw milk obtained from a major Chinese milk-producing city were analyzed for 22 steroids in four categories (glucocorticoids, androgens, estrogens, and progestogens) by HPLC-MS/MS. Only cortisol and progesterone were detected. The maximum detectable value of cortisol in raw milk in this study was lower than the MRL specified in Japan. In addition, all of the detected concentrations of cortisol were within the normal range reported in the literature. When consumption was entirely in milk, estimated dietary exposure in children (aged 1–5) corresponded to 1.48–1.60% of the ADI, using the maximum value of progesterone. Our risk assessment suggested that the detectable steroid hormones in raw milk were insufficient to cause a health risk in children.

## Figures and Tables

**Table 1:** High-performance liquid chromatography-tandem mass spectrometry (HPLC-MS/MS) conditions and internal standards for analysis.

Analyte	Parent Ion (m/z)	Daughter Ion (m/z)	Cone Voltage (V)	Collision Energy (eV)	Internal Standard
Prednisone	403.7	327.5 ^a^	18	14	Cortisol-*d*_3_
		357.2		9	
Cortisol	407.5	331.5 ^a^	25	16	Cortisol-*d*_3_
		361.7		13	
Dexamethasone	437.4	361.5 ^a^	30	16	Cortisol-*d*_3_
		391.3		12	
Fludrocortisone acetate	467.4	421.2 ^a^	20	12	Cortisol-*d*_3_
		349		24	
Beclomethasone	453.3	377.3 ^a^	20	15	Cortisol-*d*_3_
		407.3		12	
Methylprednisolone	419.7	343.6 ^a^	20	19	Cortisol-*d*_3_
		373.3		12	
Prednisolone	407.5	331.5 ^a^	25	16	Cortisol-*d*_3_
		361.7		13	
Trenbolone	271.2	253.3 ^a^	33	18	17β-boldenone-*d*_3_
		199.3		24	
Boldenone	287.6	121.0 ^a^	22	22	17β-boldenone-*d*_3_
		135		15	
Nandrolone	275.6	109.1 ^a^	35	26	17β-boldenone-*d*_3_
		257.4		15	
Testosterone	289.4	97.1 ^a^	38	22	testosterone-3,4-^13^C_2_
		109.1		20	
Methyltestosterone	303.5	109.1 ^a^	20	27	methyltestosterone-*d*_3_
		97.1		25	
Methandrostenolone	287.4	269.1 ^a^	16	11	testosterone-3,4-^13^C_2_
		159.1		21	
Stanozolol	329.5	81.1 ^a^	60	42	16β-OH-stanozolol-*d*_3_
		91.1		40	
Megestrol acetate	385.5	267.3 ^a^	30	16	megestrol acetate-*d*_3_
		325.6		16	
Chlormadinone acetate	405.4	345.6 ^a^	28	12	megestrol acetate-*d*_3_
		309.6		16	
Progesterone	315.5	97 ^a^	35	20	progesterone-*d*_9_
		297.5		35	
Estriol	287.3	145.3 ^a^	56	44	estradiol-3,4-^13^C_2_
		171.1		47	
Estradiol	271.4	183.1 ^a^	45	40	estradiol-3,4-^13^C_2_
		145.2		45	
Estrone	269.4	145.2 ^a^	49	41	estrone-2,4-*d*_2_
		159.2		41	
Diethylstilbestrol	267.3	251.3 ^a^	43	25	diethylstilbestrol-*d*_6_
		237.3		28	
Hexestrol	269.5	133.9 ^a^	30	16	dienestrol-*d*_2_
		119.1		40	

^a^: Quantitative ion.

**Table 2:** The recovery and limit of quantification (LOQ) of the method.

Analyte	LOQ (µg/kg)	% Recovery
Prednisone	0.4	73.3–81.2
Cortisol	0.4	75.4–85.7
Dexamethasone	0.4	72.5–80.3
Fludrocortisone acetate	0.4	75.2–88.1
Beclomethasone	0.4	75.4–86.8
Methylprednisolone	0.4	72.3–80.2
Prednisolone	0.4	73.5–86.1
Trenbolone	1.0	71.0–80.0
Boldenone	1.0	75.2–85.4
Nandrolone	1.0	75.4–85.1
Methandrostenolone	1.0	74.6–82.4
Testosterone	0.4	76.5–84.5
Methyltestosterone	0.4	74.1–79.0
Stanozolol	0.4	72.1–81.2
Megestrol acetate	1.0	75.0–90.2
Chlormadinone acetate	1.0	77.5–84.3
Progesterone	0.4	78.3–92.5
Estriol	1.0	77.2–79.6
Estradiol	1.0	73.4–83.1
Estrone	2.0	71.2–80.5
Diethylstilbestrol	2.0	70.3–80.6
Hexestrol	2.0	75.2–82.7

**Table 3:** The presence of steroids in raw milk.

Analyte	Positive Samples n (%)	Steroids Content (µg/kg)		
		Maximum	Minimum	Mean ± SD
Prednisone	0 (0)		-	
Cortisol	24 (12.3)	0.94	0.44	0.61 ± 0.13
Dexamethasone	0 (0)		-	
Fludrocortisone acetate	0 (0)		-	
Beclomethasone	0 (0)		-	
Methylprednisolone	0 (0)		-	
Prednisolone	0 (0)		-	
Trenbolone	0 (0)		-	
Boldenone	0 (0)		-	
Nandrolone	0 (0)		-	
Methandrostenolone	0 (0)		-	
Testosterone	0 (0)		-	
Methyltestosterone	0 (0)		-	
Stanozolol	0 (0)		-	
Megestrol acetate	0 (0)		-	
Chlormadinone acetate	0 (0)		-	
Progesterone	165 (84.6)	9.04	2.12	5.12 ± 1.41
Estriol	0 (0)		-	
Estradiol	0 (0)		-	
Estrone	0 (0)		-	
Diethylstilbestrol	0 (0)		-	
Hexestrol	0 (0)		-	

**Table 4:** The maximum residue limit for hormones in raw milk in China, the European Union (EU), the Codex Alimentarius Commission (CAC), and Japan.

Category	Name	Maximum Residue Limit (µg/kg)			
		China	EU	CAC	Japan
Glucocorticoids	Betamethasone	0.3 ^a^	0.3 ^a^		0.3 ^a^
	Dexamethasone	0.3 ^a^	0.3 ^a^	0.3 ^a^	20 ^a^
	Cortisol				10 ^a^
	Prednisolone		6 ^a^		0.7 ^a^
	Methylprednisolone				10 ^a^
Progestogens	Altrenogest				3 ^a^
	Chlormadinone		2.5 ^a^		3 ^a^
	Norgestomet				0.1 ^a^
	Megestrol Acetate	N.D. ^b^			
Androgens	Crostebol				0.5 ^a^
	Methyltestosterone	N.D. ^b^			
	Testosterone Propionate	N.D. ^b^			
	Trenbolone	N.D. ^b^			
	Trenbolone Acetate				N.D. ^b^
	Nandrolone Phenylpropionate	N.D. ^b^			
Estrogens	Diethylstilbestrol	N.D. ^b^			N.D. ^b^
	Estradiol Benzoate	N.D. ^b^			

^a^ Maximum residue limit in the standard; ^b^ N.D.: not to be detected in the standard.

**Table 5:** Estimated dietary exposure of children aged between one and five years old to progesterone in raw milk, using the mean concentration.

Population Group		Mean Concentration in Milk (μg/kg)	Body Weight (kg) [23]	Ingestion Rate (g) [21]	Exposure Level (μg/kg body weight/day)	Contribution to the ADI (%)
Gender	Age					
Boys ^a^	1	5.12	9.65	475	0.252	0.84
	2		12.15	344	0.145	0.48
	3		14.34	347	0.124	0.41
	4		16.34	328	0.103	0.34
	5		18.34	330	0.092	0.31
Girls ^b^	1		8.95	475	0.271	0.90
	2		11.48	344	0.153	0.51
	3		13.85	347	0.128	0.43
	4		16.07	328	0.104	0.35
	5		18.22	330	0.093	0.31

**Table 6:** Estimated dietary exposure to progesterone in raw milk in children aged between one and five years, using the maximum concentration.

Population Group		Maximum Concentration in Milk (μg/kg)	Body Weight (kg) [23]	Quantity Ingested (g) [21]	Exposure Level (μg/kg body weight/day)	Contribution to the ADI (%)
Gender	Age					
Boys	1	9.04	9.65	475	0.445	1.48
	2		12.15	344	0.256	0.85
	3		14.34	347	0.219	0.73
	4		16.34	328	0.181	0.60
	5		18.34	330	0.163	0.54
Girls	1		8.95	475	0.480	1.60
	2		11.48	344	0.271	0.90
	3		13.85	347	0.226	0.75
	4		16.07	328	0.184	0.61
	5		18.22	330	0.164	0.55
